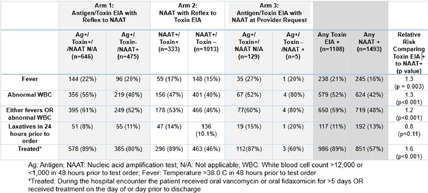# Clostridioides difficile Testing Strategies: Insights into Positivity Rates, Systemic Signs, and Treatment Patterns

**DOI:** 10.1017/ash.2025.199

**Published:** 2025-09-24

**Authors:** Katie Passaretti, Shelley Kester, Jessica Layell, Elizabeth Palavecino, Jackson Morton, Werner Bischoff, Anupama Neelakanta

**Affiliations:** 1 Atrium Health Wake Forest; 2 Advocate Health; 3 Atrium Health; 4 Wake Forest University School of Medicine

## Abstract

**Background:** The diagnostic approach for Clostridioides difficile infection (CDI) significantly influences treatment and resource utilization. This study compares clinical characteristics and treatment choices based on three testing algorithms combining antigen and toxin enzyme immunoassay (EIA) tests and nucleic acid amplification tests (NAAT). **Methods:** We performed a retrospective study of patients tested for CDI between August 2022 and November 2024 in a large health system where multiple CDI testing algorithms are utilized: (arm 1) antigen and toxin EIA with automatic reflex to NAAT if discrepant results; (arm 2) NAAT with automatic reflex to toxin EIA if NAAT positive; and (arm 3) antigen and toxin EIA with NAAT at provider request with approval by Antimicrobial Stewardship. The last step in the testing algorithms determined whether the result was considered positive. We determined positivity rate by algorithm results and compared clinical variables including fever (temperature > 38.0° C) or abnormal white blood cell (WBC) count (12,000) within 48 hours prior to test order, laxative use within 24 hours prior to test order and treatment rates between those who tested toxin positive by EIA and those who tested toxin positive by NAAT only. Treatment was defined as receiving oral vancomycin or fidaxomicin for more than 5 days OR receiving those medications on the day prior to or day of discharge. **Results:** A total of 16,555 patients were tested. Overall algorithm positivity rate was highest in the EIA with reflex to NAAT (arm 1) at 13.7% compared to 5.7% for arm 2 (NAAT with reflex to toxin EIA) and 5.1% for arm 3 (EIA with NAAT at Provider Request). Toxin EIA positive patients were 1.2 times more likely than NAAT positive patients to display fever or abnormal WBC in the 48 hours prior to test order (p < 0 .001). Toxin EIA positive patients were less likely to receive laxatives compared to NAAT only positive patients. (p=0.11). Among toxin EIA positive cases, 89% received treatment compared to 57% in toxin NAAT only positive cases (p < 0 .001). 46% of patients who tested NAAT positive with a subsequently negative toxin EIA were treated. **Conclusion:** Patients with toxin EIA positive tests were more likely to exhibit systemic signs of infection and were treated at higher rates compared to NAAT-positive cases. While NAAT-based testing identified additional cases, many may reflect colonization. Treatment of toxin NAAT positive/toxin EIA negative patients was common highlighting opportunities for diagnostic stewardship.

**Figure:**
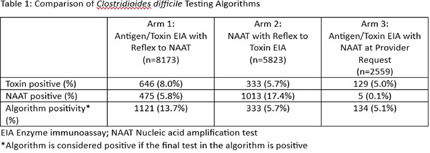

**Figure:**